# Young Adults with Inflammatory Bowel Disease in the US Experience Gaps in Healthcare Access and Financial Stress: Additional Findings from a Recent Survey by the Crohn’s & Colitis Foundation

**DOI:** 10.1093/crocol/otaf066

**Published:** 2026-01-08

**Authors:** Ross M Maltz, Ariel A Jordan, Shubha Bhat, Mary Harkins-Schwarz, Orna G Ehrlich

**Affiliations:** Division of Pediatric Gastroenterology, Hepatology and Nutrition, Nationwide Children’s Hospital, Columbus, OH 43205, United States; Department of Pediatrics, The Ohio State Wexner Medical Center, Columbus, OH 43210, United States; Department of Internal Medicine, University of Michigan, Ann Arbor, MI 48104, United States; Departments of Pharmacy and Digestive Disease Institute, Cleveland Clinic Foundation, Cleveland, OH 44195, United States; Crohn’s & Colitis Foundation, New York, NY, United States; Crohn’s & Colitis Foundation, New York, NY, United States

**Keywords:** inflammatory bowel disease, insurance, barriers to care, Crohn’s disease, ulcerative colitis

## Abstract

**Background:**

Young adults with chronic conditions are at increased risk for active disease and for poor adherence, resulting in increased emergency department utilization and hospitalizations. We aimed to evaluate whether young adults with inflammatory bowel disease (IBD) experienced more healthcare access challenges and financial distress compared to adults and pediatric patients.

**Methods:**

A survey developed by the Crohn’s & Colitis Foundation was electronically disseminated to adults with IBD and caregivers across the United States to assess healthcare access and financial challenges. Analyses were completed to evaluate differences across patient age groups (caregivers on behalf of pediatric patients <18 years, young adults 18-25 years, and adults 26-64 years) using Chi square or Fisher’s exact tests.

**Results:**

Of 1781 respondents (77% adult, 12.9% young adult, and 10.1% pediatric caregivers), there were no significant differences between groups in obtaining insurance approval and experiencing adverse health events due to treatment delays. However, young adults were more likely to experience step therapy mandates and less likely to know what questions to ask their insurer if experiencing coverage problems compared to adult and pediatric patients’ caregivers. Additionally, young adult patients were more likely to take on an extra job or work more hours to afford their healthcare or insurance costs related to IBD.

**Conclusion:**

Additional analysis from the Crohn’s & Colitis Foundation access assessment highlights the need for more resources and support in navigating healthcare access for young adult patients with IBD.

## Introduction

Patients with inflammatory bowel disease (IBD) in the United States continue to face persistent challenges with healthcare access, high treatment costs, and insurance barriers, as highlighted in a recent publication.^1^ Inflammatory bowel disease-related medications are the primary driver of costs for IBD care.^2^ Insurance companies often require prior authorizations (PA) and step therapy mandates (ie, mandating a trial of a less expensive medication instead of the physician-prescribed medication), which can cause treatment delays and worsen health outcomes. These processes contribute to financial strain, which further impacts health outcomes.^1^

Young adults with IBD face additional difficulties when transitioning from pediatric to adult care. This period is associated with lapses in routine care, poor adherence, increased emergency department utilization, and hospitalizations.^3^^,^^4^ We aimed to evaluate whether young adults with IBD had greater barriers in access and financial distress compared to adult and pediatric patients.

## Methods

The Crohn’s & Colitis Foundation conducted a US-based survey among adults with IBD and caregivers on behalf of pediatric patients. Inflammatory bowel disease diagnosis was self reported. Detailed methodology, including survey design, participant recruitment, and data collection procedures, has been previously described.^1^ The survey included questions on medication access, experience with step therapy mandates, financial barriers to obtaining medications, and disease and demographic characteristics. The study was reviewed by the Advarra Institutional Review Board (IRB) and deemed exempt from IRB oversight.

Additional analyses were conducted to examine variations across patient age groups (pediatric caregiver [<18 years], young adult [18-25 years], and adult [26-64 years]). We assessed whether there were differences in key outcomes based on age groups, specifically medication access/coverage, confidence with navigating insurance barriers, experienced step therapy mandates, and financial barriers to care.^1^ To determine statistically significant differences across age groups for the hypothesis of interest, Chi square or Fisher’s exact tests were used. All analyses were conducted using GraphPad Prism version 10.3.1 (GraphPad Software, San Diego, CA). A *P*-value <.05 indicated statistical significance.

## Results

Of 1781 respondents, 1372 (77%) were adults, 229 (12.9%) were young adults, and 180 (10.1%) were pediatric caregivers (Table 1). Most respondents were female in all age groups. Ethnicity and race varied significantly, with young adults reporting the highest percentage of Hispanic respondents (7.7%) and the lowest percentage of Black respondents (1.4%). Respondents had a higher percentage of health insurance compared to the US population.^5^^,^^6^ The young adult group had the lowest rate of health insurance coverage (96%), which is similar to the US population overall for this age group.^7^ A higher percentage of pediatric and young adults receive IBD care in an academic setting compared to adults.

**Table 1.** Demographic characteristics and experiences of respondents IBD health access assessment by age groups (total *N* = 1781).

Age ranges	Pediatric caregiver 0-17 years old *N* = 180 (10%)	Young adults 18-25 years old *N* = 229 (13%)	Adults 26-64 years old *N* = 1372 (77%)	*P-*value
**Gender**				**<.001**
** Female**	102 (57.3%)	136 (59.4%)	999 (72.9%)	
** Male**	73 (41%)	85 (37.1%)	339 (24.7%)	
** Transgender/use a different term**	3 (1.7%)	8 (3.5%)	33 (2.4%)	
**Ethnicity**				**.04**
** Hispanic**	4 (2.3%)	17 (7.7%)	58 (4.4%)	
**Race**				**.005**
** White**	151 (86.3%)	184 (83.3%)	1,155 (87.2%)	
** Black**	9 (5.1%)	3 (1.4%)	59 (4.5%)	
** Other**	11 (6.3%)	17 (7.7%)	53 (4%)	
**Insurance insured**	178 (99.4%)	212 (95.9%)	1,320 (97.7%)	**.005**
**Poverty**				.15
** Not concentrated poverty (19.9% or less)**	152 (95.6%)	197 (95.2%)	1,171 (92.1%)	
** Concentrated poverty (20.0% or more)**	7 (4.4%)	10 (4.8%)	101 (7.9%)	
**Receives IBD care in an academic setting**				.05
** Yes**	76 (44.2%)	97 (44.1%)	483 (37.3%)	
** No**	96 (55.8%)	123 (55.9%)	813 (62.7%)	
**Disease type**				.07
** Crohn’s disease**	126 (70%)	162 (70.7%)	896 (65.3%)	
** Ulcerative colitis**	44 (24.4%)	64 (27.9%)	419 (30.5%)	
** IBD—unclassified**	8 (4.4%)	3 (1.3%)	31 (2.3%)	
** Unknown or undetermined**	2 (1.1%)	0 (0%)	26 (1.9%)	
***Experiences of respondents prescribed any medication for their IBD in prior 12 months***				
**Patient use of biologic therapy or small molecule to treat their IBD**	155 (90.1%)	189 (85.5%)	944 (76.4%)	**<.01**
**Patient experienced issues accessing their medications due to insurance**				.08
** No issues**	69 (40.8%)	68 (31.5%)	475 (39.2%)	
** Experienced issues**	100 (59.2%)	148 (68.5%)	736 (60.8%)	
**Patient experienced a delay in receiving their medication due to insurance**				.23
** No or minimal**	148 (85.5%)	175 (79.9%)	979 (80.1%)	
** Experience 1 or more month delay**	25 (14.5%)	44 (20.1%)	243 (19.9%)	
**Patient experienced adverse health events due to not taking their IBD medication as prescribed due to insurance barriers**				.16
** Yes**	19 (23.2%)	29 (21.6%)	208 (28.8%)	
** No**	63 (76.8%)	105 (78.4%)	513 (71.2%)	
**Patient experienced effects on their daily activities due to not taking their IBD medication as prescribed due to insurance barriers**				.16
** No effects**	19 (47.5%)	29 (35.8%)	208 (47.2%)	
** Experienced effects on daily activities**	21 (52.5%)	52 (64.2%)	233 (52.8%)	
**Patient experienced step therapy mandates in the past 12 months**	34 (20.4%)	73 (34.8%)	318 (27.2%)	**.007**
**Patient experienced a significant delay or denial in test/treatment due to insurance in the past 12 months**	48 (27.7%)	61 (28.6 %)	251 (20.9%)	**.01**
***Experiences of respondents with financial barriers to related to their IBD in prior 12 months***				
**Patient experienced 1 or more financial barriers to pay IBD healthcare or insurance costs**	72 (40%)	101 (44.1%)	647 (47.2%)	.38
**Had trouble paying or were unable to pay medical bills related to IBD in the past 12 months**	38 (21.2%)	60 (26.4%)	407 (29.7%)	**.04**
**Borrowed money from friend or family**	9 (5%)	32 (14.2%)	193 (14.2%)	**.02**
**Took out any type of loan or sought aid from charity**	4 (2.2%)	13 (5.7%)	78 (5.7%)	.31
**Took money out of retirement, college, or savings**	18 (10.2%)	35 (15.6%)	187 (13.8%)	.34
**Took an extra job or worked more hours**	20 (11.3%)	40 (17.6%)	153 (11.3%)	**.03**
**Cut back on food, clothing or basic household items**	39 (21.9%)	45 (19.8%)	352 (25.8%)	.17
**Increased credit card debt**	29 (16.2%)	49 (21.6%)	362 (26.6%)	**.03**
**Put off vacations or major household purchases**	57 (32%)	71 (31.3%)	485 (35.6%)	.67

Abbreviation: IBD, inflammatory bowel disease.

The adult group had the lowest utilization rate of biologic/small molecule therapy to treat their IBD compared to the pediatric caregivers and young adult groups (Table 1). There were no significant differences among the groups regarding medication access issues secondary to health insurance. Among respondents who experienced significant delays, there was no difference between groups as related to adverse health events or negative effects on daily activities (eg, inability to work, attend school). However, the young adult group (35%) was more likely to experience step therapy mandates compared to adult (27%) and pediatric caregivers (20%) groups.

**Figure 1.**
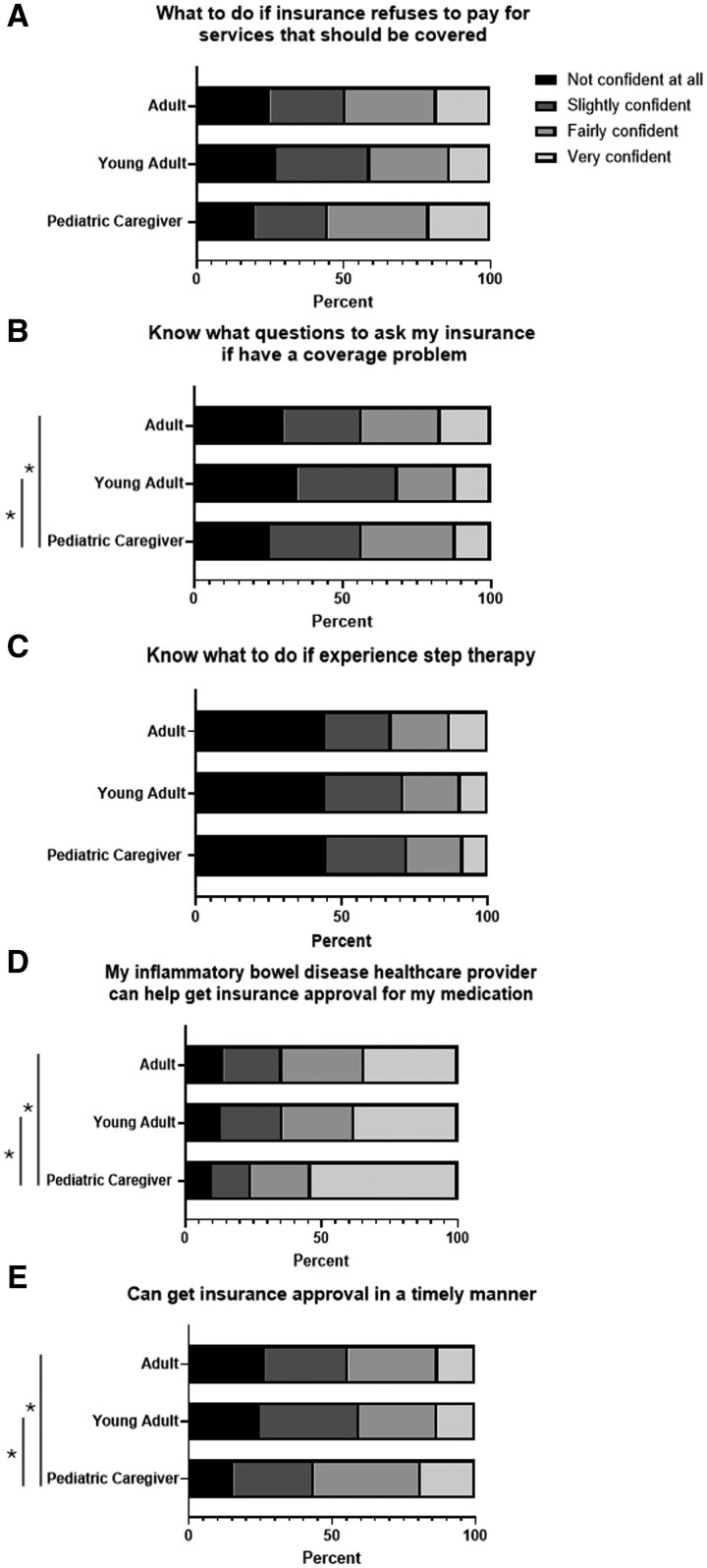
Confidence in navigating insurance barriers by age groups: *P < .05. A. What to do if insurance refuses to pay for services that should be covered. B. Know what questions to ask my insurance if have a coverage problem. C. Know what to do if experience step therapy. D. My inflammatory bowel disease healthcare provider can help get insurance approval for my medication. E. Can get insurance approval in a timely manner.

The young adult group demonstrated lower confidence in knowing which questions to ask their insurance provider when experiencing coverage issues compared to the pediatric caregivers’ group (35% vs 25% were “not confident at all”) (Figure 1). Additionally, the young adult and adult groups were less assured in their healthcare provider’s ability to obtain timely insurance medication approval compared to the pediatric group. Across all three groups, nearly 50% expressed “not/slightly confident” in navigating situations when insurance refused to pay for services or mandated step therapy.

All age groups reported high rates (40%-47%) of experiencing one or more financial barriers related to IBD care. Adults (30%) were more likely to face difficulties paying IBD-related medical bills compared to the pediatric caregivers’ group (21%) (*P* = .02). Both young adults (14%) and adults (14%) were more likely to borrow money from friends or family to pay for IBD-related medical bills than the pediatric caregivers’ group (5%) (*P* < .01). Additionally, young adults (17.6%) were more likely to work an extra job or work more hours to pay for IBD-related medical bills compared to pediatric caregivers (11%) and adult (11%) groups (*P* < .05).

## Discussion

As observed in prior Crohn’s & Colitis Foundation access assessments, IBD patients still face substantial financial burdens and access issues in the United States.^1^^,^^8^ These issues persist due to insurance processes, such as PA and step therapy mandates. These barriers continue to delay timely care and hinder access to essential medications and tests, leading to financial distress, adverse health outcomes, and negative impacts on quality of life for patients.^1^ This becomes increasingly important for young adults making the transition from pediatric to adult care as this can lead to lapses in routine care, adherence issues, and ultimately disease flares and hospitalizations.^3^^,^^4^

Young adults are at an age where they often begin to manage their own health care and advocate for themselves. Many experience moving off their parents’ health insurance to a school or employer health insurance. This period of adjustment may influence why young adults were more significantly impacted by step therapy mandates and reported lower confidence in navigating insurance processes. These financial hardships may compel them to work an extra job or more hours to manage medical debt. This underscores the need for additional resources, education, and support to assist young adults in overcoming healthcare access and financial challenges especially when they transition into adult care.

One study limitation is that respondents may not be representative of the broader US IBD population, likely due to the survey’s recruitment methods. Additionally, the survey did not assess whether young adults were dependents or primary insurance subscribers, or which care setting (academic or community) they used. Future research should further examine health care access during the transition to adult care, particularly as patients begin managing their own insurance coverage.

## Author Contributions

Study conceptualization, methodology, writing, reviewing, and editing were completed by all of the authors. Ross M. Maltz completed the analysis.

## Funding

This study was funded by the Crohn’s & Colitis Foundation.

## Data Availability

Data is not publicly available. Please contact the authors for access to the data.

## References

[1] Jordan AA , Bhat S, Ali T, et al. Healthcare access for patients with inflammatory bowel disease in the United States: a survey by the Crohn’s & Colitis Foundation. Inflamm. Bowel Dis. 2025;31:1819-1832. 10.1093/ibd/izae237 39377748 PMC12235136

[2] Kahn-Boesel O , Cautha S, Ufere NN, Ananthakrishnan AN, Kochar B. A narrative review of financial burden, distress, and toxicity of inflammatory bowel diseases in the United States. Am J Gastroenterol. 2023;118:1545-1553. 37224301 10.14309/ajg.0000000000002345

[3] Cole R , Ashok D, Razack A, Azaz A, Sebastian S. Evaluation of outcomes in adolescent inflammatory bowel disease patients following transfer from pediatric to adult health care services: Case for transition. J Adolesc Health. 2015;57:212-217. 26206442 10.1016/j.jadohealth.2015.04.012

[4] Pearlstein H , Bricker J, Michel HK, et al. Predicting suboptimal transitions in adolescents with inflammatory bowel disease. J Pediatr Gastroenterol Nutr. 2021;72:563-568. 33264185 10.1097/MPG.0000000000003013

[5] National Center for Health Statistics. Percentage of being uninsured at the time of interview for children under age 18 years, United States, 2019—2024. National Health Interview Survey. Generated interactively: (2025). https://wwwn.cdc.gov/NHISDataQueryTool/SHS_adult/index.html

[6] National Center for Health Statistics. Percentage of being uninsured at the time of interview for adults aged 18-64, United States, 2019—2024. National Health Interview Survey. Generated interactively: (2025). https://wwwn.cdc.gov/NHISDataQueryTool/SHS_child/index.html

[7] U.S. Census Bureau, Current Population Survey, 2023 and 2024 Annual Social and Economic Supplements.

[8] Rubin DT , Feld LD, Goeppinger SR, et al. The Crohn’s and Colitis Foundation of America survey of inflammatory bowel disease patient health care access. Inflamm. Bowel Dis. 2017;23:224-232. 27997434 10.1097/MIB.0000000000000994

